# The continuous and discrete molecular orbital x-ray bands from Xe^q+^ (12≤q≤29) +Zn collisions

**DOI:** 10.1038/srep30644

**Published:** 2016-07-29

**Authors:** Yipan Guo, Zhihu Yang, Bitao Hu, Xiangli Wang, Zhangyong Song, Qiumei Xu, Boli Zhang, Jing Chen, Bian Yang, Jie Yang

**Affiliations:** 1 Institute of Modern Physics, Chinese Academy of Sciences, Lanzhou 730000, P.R. China; 2 University of Chinese Academy of Sciences, Beijing 100049, P.R. China; 3 School of Nuclear Science and Technology, Lanzhou University, Lanzhou 730000, P.R. China; 4 College of Physics and Electronic Engineering, Northwest Normal University, Lanzhou 730070, P.R. China

## Abstract

In this paper, the x-ray emissions are measured by the interaction of 1500–3500 keV Xe^q+^ (q = 12, 15, 17, 19, 21, 23, 26 and 29) ions with Zn target. When q < 29, we observe Ll, Lα, Lβ_1_, Lβ_2_ and Lγ characteristic x-rays from Xe^q+^ ions and a broad M-shell molecular orbital (MO) x-ray band from the transient quasi-molecular levels. It is found that their yields quickly increase with different rates as the incident energy increases. Besides, the widths of the broad MO x-ray bands are about 0.9–1.32 keV over the energy range studied and are proportional to v^1/2^ (v = projectile velocity). Most remarkably, when the projectile charge state is 29, the broad x-ray band separates into several narrow discrete spectra, which was never observed before in this field.

Research on charge-exchange processes during ion-atom collisions has attracted considerable interest for recent decades^1^,^2^,^3^,^4^,^5^. For slow highly charged ion interactions with the target, quasi-molecular states that are formed during collisions have proved to be successful in qualitative describing electron-vacancy exchange processes^6^,^7^,^8^,^9^,^10^,^11^,^12^. In this frame, as the collision partners approach each other, the atomic orbitals evolve into the molecular orbitals and some atomic levels may turn into two or more molecular levels^6^,^7^.

When a vacancy participates in such an interaction, an electron in a low-lying level may be promoted to the vacant orbital through so-called electron promotion^1^,^6^,^7^. For example, most K-shell vacancy productions are attributed to the promotion of 2pσ → 2pπ at slow collisions. 2pσ and 2pπ molecular orbitals are from the low-Z partner 1s and high-Z partner 2p atomic orbitals, respectively. This mechanism has been used to interpret a wealth of experimental results, such as the origin of characteristic lines, the energy level matching effect, the charge state effect and the atomic number effect, among others^1^,^9^,^10^,^12^,^13^,^14^. Alternatively, the vacancy brought into the quasi-molecular system also is possible to be filled by an electron occupying a high-lying level, accompanied by molecular orbital (MO) x-ray emission, i.e., MO transition. Saris *et al*. first reported this phenomenon by measuring x-ray emissions from Ar ions of energy 70–600 keV impinging on C, Al, Si and Fe targets^11^. In their experiment, they observed a broad MO x-ray band, which was attributed to the radiative filling of a vacancy in the 2pπ level of the Ar-Ar system during the collision. To date, several experimental investigations of MO x-rays have been reported^15^,^16^,^17^,^18^.

As we know, the formation of the quasi-molecule may allow us to understand the properties of the super-heavy atoms in advance and x-ray emission can provide some deeper insights on the dynamical process of its formation. This is why a lot of experiments were devoted to x-ray emissions in slow collisions. Unfortunately, the molecular orbitals are difficult to calculate, especially for higher shells and multi-electrons systems^19^,^20^,^21^. Therefore, the precise theoretical calculations in electron promotion and MO transition processes are still lacking for slow highly charged ion-atom collisions. More experiments are necessary for its understanding.

Although, under certain conditions, both the processes of electron promotion and MO transition can occur in the same ion-atom collision and there were many experiments focusing on the collision between slow highly charged ion and atom, these two processes have never been observed and studied simultaneously in experiment up to now.

In order to check whether these two processes can happen in the same slow ion-atom collision and understand how they happen, in this paper, the collisions between 1500–3500 keV Xe^q+^ ions (q = 12–29) with Zn target are studied by measuring the x-rays produced from it.

## Experiment method

The experiment was performed using the 320 kV high voltage platform of an all permanent Electron Cyclotron Resonance Ion Source (ECRIS) at the Institute of Modern Physics (IMP), Chinese Academy of Sciences (CAS). It was designed to be operated at 14.5 GHz with the purpose of producing medium charge state and high charge state gaseous and metallic ion beams^22^. Xe^q+^ ions of charge states 12, 15, 17, 19, 21, 23, 26 and 29 in the energy range of 1500 to 3500 keV were provided by the ECRIS. The essentials of the experimental apparatus were described in detail elsewhere^8^. The Xe^q+^ ion beam, extracted and selected by a 90° analyzing magnet, was first highly collimated by two sets of four jaw slits and used to bombard the surface of a Zn target at 45°. The target used in our experiment, with a purity of 99.99% and a polished surface, had a thickness of 50 μm and a size of 20 × 21 mm^2^. The emitted x-rays were counted by a Si(Li) detector placed at 90° relative to the incident direction of the beams. The Si (Li) detector had an energy resolution of 165 eV at 5.9 keV and a detection range of 1–60 keV. The energy calibration of the detector was made by using radioactive sources ^241^Am and ^55^Fe. The pressure of the target chamber was maintained at 2 × 10^−8^ mbar. In order to reduce the statistical error, each measurement was taken for 3000 seconds.

## Results and Discussion

**Figure 1:**
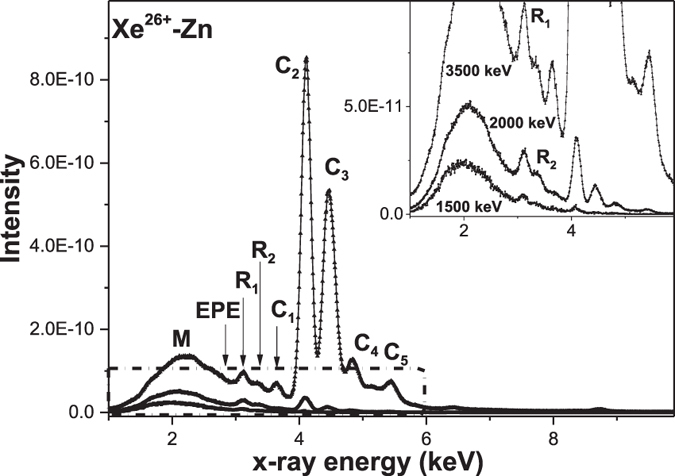
Typical x-ray spectra from collisions of Xe^q <29+^ ions with a Zn target at 1500 keV, 2000 keV and 3500 keV (from bottom to top). The insert graph shows a zoomed-in view of the x-ray spectral structure in the dotted box. M: MO x-ray band; EPE: end point energy; R_1_ and R_2_: radiative Auger effect peaks; C_1_, C_2_, C_3_, C_4_, and C_5_: Ll, Lα, Lβ_1_, Lβ_2_ and Lγ x-ray peaks, respectively, of Xe^q+^ ions.

**Figure 2:**
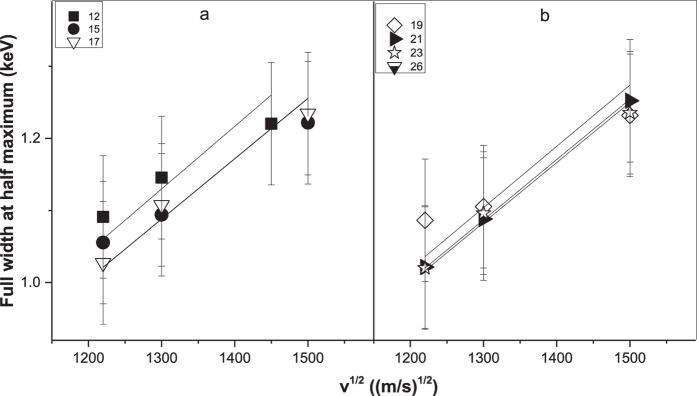
Full width at half maximum (FWHM) of the M band for Xe^12, 15, 17+^ (**a**) and Xe^19, 21, 23, 26+^ (**b**) vs v^1/2^. The solid lines represent the fitting results obtained using FWHM = kv^1/2^.

Typical x-ray spectra obtained with Xe^q<29+^ ions incident upon the target at different incoming energies are shown in Fig. 1. All spectra have been normalized by incident particle number. From top to bottom, the spectra were induced by 3500, 2000 and 1500 keV Xe^q+^ ions, respectively. To clearly show the low energy excited spectral structure, a zoomed-in view of the spectra in the dotted box is presented in the inset of Fig. 1. Obviously, in Fig. 1, every measured spectral structure can be divided into two components, namely, a non-characteristic x-ray band M and the characteristic x-ray lines C_1_, C_2_, C_3_, C_4_, and C_5_, by their full widths at half-maximum (FWHM). FWHMs of the x-ray bands are approximately 0.90–1.32 keV, and their end point energy of 2.7 keV agrees well with the energy of the M-shell x-rays in the united Po atom limit^23^. According to the Fano-Lichten description of heavy-ion collisions, we know that radiative decay of the vacancies can occur during the MO formation process, and the emitted x-ray spectra have a broad FWHM and end point energy (EPE) that matches with the united atom (UA) characteristic x-ray energy. Hence, in our results, the pronounced non characteristic M bands are expected as M-shell MO x-rays of united Po atoms. Meanwhile, the peaks, marked as C_1_, C_2_, C_3_, C_4_, and C_5_, which respectively peak at 3.6, 4.0, 4.4, 4.8 and 5.4 keV, are identified as Ll, Lα, Lβ_1_, Lβ_2_ and Lγ characteristic x-ray of the Xe^q+^ ions^24^. From the above figure, as the energy of the Xe^q+^ ions increasing, a large increase is simultaneously observed in the yields of the characteristic and non-characteristic x-rays. Moreover, the FWHM of the MO x-ray spectrum is also increasing with increasing energy. In addition, plots of the M band FWHM versus v^1/2^ for different charge states are shown in Fig. 2. From this figure, one can see that the FWHM of the band M is proportional to the square root of the projectile velocity. This finding is consistent with that predicted by Betz *et al*.^25^,^26^. But in our experiment, the slope is different from that given by Betz *et al*. because they focused on the K-shell measurement.

**Figure 3:**
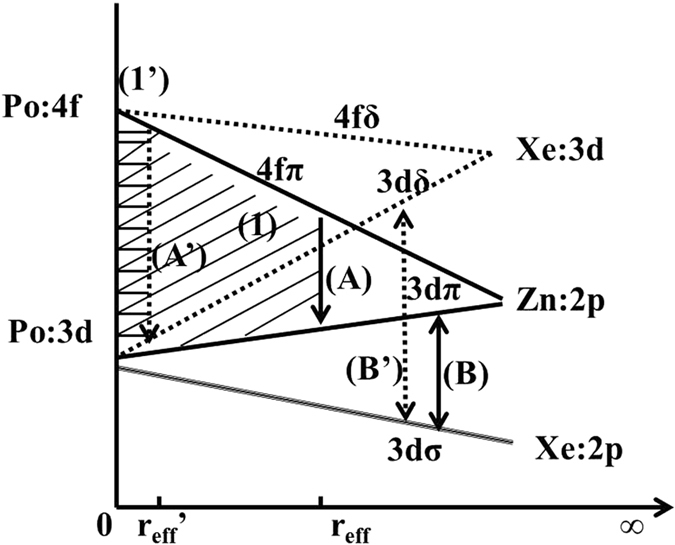
Molecular correlation diagram (a qualitative estimate of the Xe + Zn molecular levels). (1) and (1’): MO transition region; (A) and (B): MO transition and electron promotion for a Xe^q<29+^+ Zn atom collision; (A’) and (B’): MO transition and electron promotion for a Xe ^29+^+ Zn atom collision; r_eff_ (r’_eff_): effective range of MO transition induced by Xe^q<29+^ (Xe^29+^).

To understand the origin of the M band and the Xe^q+^ L-shell characteristic lines, an approximate correlation diagram representing the Xe-Zn molecule based on MO theory is presented in Fig. 3 7.

To be specific, in this theory, as the inter-nuclear distance shrinks, the target 2p orbital evolves to 2pπ, 3dπ and 4fπ MOs, of which the unoccupied 3dπ is filled by the 4fπ to generate MO x-rays (denoted by arrow (A) in Fig. 3). When the vacancies decay at the closet distance, the high energy limit, i.e., the tail of the MO line, occurs; this energy is equal to that of the M x-rays of the united Xe-Zn atom (Po, Z = 84), in our results. After collision, a broad molecular x-ray spectrum is formed. Meanwhile, during the interaction, the electron promotion process (denoted by arrow (B)) is also expected. The 2p electron of Xe^q+^ is promoted by 3dσ-3dπ rotational coupling^1^,^6^,^7^. After collision, the Xe^q+^ ions carry 2p vacancies, whose radiative decay leads to the emission of Xe^q+^ L-shell x-rays.

Obviously, for the 4fπ → 3dπ and 3dσ → 3dπ transitions to occur, the target atom must carry 2p vacancies. As described by W. Meyerhof *et al*.^27^,^28^, these 2p vacancies could be created through a one-step or a two-step process in the first collision, and they may then be carried into the second collision and participate in subsequent interactions. Here, in our paper, we also suggest that the 2p vacancies have been created before the process described in Fig. 3, as the production of both the MO and characteristic x-rays need the 2p vacancies, and we do not look further into how they are produced.

According to the classical over-barrier (COB) model^29^,^30^, when the distance between the incident ion and the solid surface reaches a critical distance 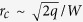, electrons can be resonantly captured from the surface into projectile highly excited states 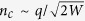, where *W* is the work function of the metal surface (in atomic units). The charge transfer process continues until the ion is almost fully neutralized and a hollow atom/ion (HA) is formed. The potential energy of the ion is mainly released into the metal surface. The time τ_c_ required for the HA to arrive at the surface is approximately *r*_*c*_/*v.* In the experiments reported here, *W*(*Zn*) is 0.123 a.u. and v is 1.48~2.26 × 10^6^ m/s; therefore, for q = 12, r_c_ ~ 39.83 a.u., n_c_ ~ 24, and τ_c_ = 0.93~1.42 × 10^−15^ s and for q = 26, r_c_ ~ 58.63 a.u., n_c_ ~ 54, and τ_c_ = 1.37~2.10 × 10^−15^ s. That is, in our experiment, the time required for a HA to arrive at the target surface is about 10^−15^ s. As we know, the time for a HA to decay to the ground state is about 10^−13^–10^−14^ s, which is larger than τ_c_. This indicates that the x-rays from the HA decays are ignorable. When the HA impacts onto the target surface, its electrons in high Rydberg states are mostly stripped off. Subsequently, only when the ions enter into the target (forming a new impact hollow atom/ion), electron-vacancy exchange process described by Fig. 3 occurs. This analysis proves from another perspective that the MO x-rays and characteristic x-rays observed in the present experiment originate mainly from below-surface.

From Fig. 3, one can see that the target 2p vacancy is shared by the 4fπ → 3dπ and 3dσ → 3dπ transition processes. This leads to competition between the production of MO x-rays and characteristic x-rays, which is also reflected in Fig. 1. This competition can be observed for all projectile charge states. From the correlation diagram, it is clearly evident that the coupling process requires a Zn 2p vacancy (3dπ orbital) and a Xe 2p electron (3dσ orbital), whereas the 2p electron (4fπ orbital) and the 2p vacancy (3dπ orbital) of the MO transition both originate from the Zn atom. Thus, the x-ray yields and the competition between them are primarily driven by the number of Zn 2p electrons. As the incident energy increase, the number of Zn 2p vacancies also increase, which leads to an increase in the production of characteristic x-rays and MO x-rays. However, the consequent decrease in the number of Zn 2p electrons causes the growth of the MO x-ray production to be slower than that of the characteristic photon production. As a result, the ratio of MO x-ray cross sections (σ_MO_) to characteristic x-ray cross sections (σ_cha_) is decreasing with increasing incident energy. This nicely explains the measured dependence of σ_MO_/σ_cha_ on the projectile energy in our experiment, as shown in Fig. 1. For greater clarity, we present the following quantitative analysis.

Assuming that the Xe^q+^ ions slow down along a straight trajectory and emit x-rays isotopically and neglecting the energy loss straggling, the x-ray production cross sections σ could be extracted from the x-ray yield Y(E) using the standard formula^1^:


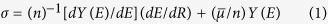



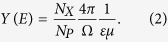


**Figure 4:**
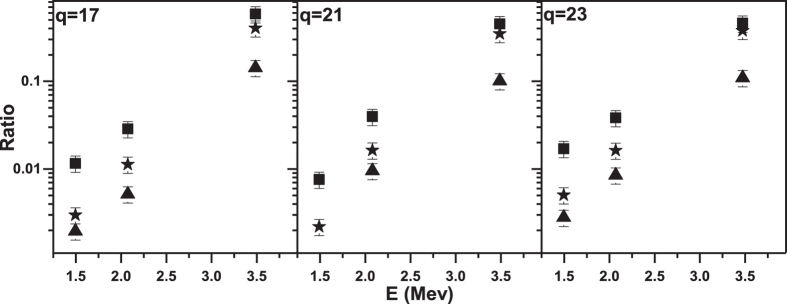
Variations in the ratios σ_Lα_/σ_MO_, σ_Lβ1_/σ_MO_, σ_Lβ2_/σ_MO_ as functions of the incident energy for charge states of 17, 21 and 23. Square: σ_Lα_/σ_MO_; Star: σ_Lβ1_/σ_MO_; Triangle: σ_Lβ2_/σ_MO_.

The quantity Y(E) represents the x-ray yield; N_x_ is the total detected x-ray counts for each spectrum; N_p_ is the total number of incident particles; Ω expresses the solid angle seen by the detector from the target, which is 23.8 msr in the present experiment; ε expresses the detector efficiency of Si(Li) detector calculated using manufacture’s specifications; 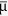 is the self-absorption coefficient of the target for its own x-rays and is acquired from NIST; μ is the photon filter transmission coefficient in 2 cm air and a 50 μm beryllium window^31^; n refers the target atom density; dE/dR represents the stopping power for incoming ions in the target, which is calculated using the SRIM-2010 program^32^. The uncertainty in the x-ray production cross sections including statistical and systematic errors is about 15%, which results in a total error of 21% in the ratio of σ_MO_/σ_cha_. Figure 4 shows the ratio of Lα x-ray cross sections (σ_Lα_) to MO x-ray cross sections (σ_Lα_/σ_MO_), _Lβ1_ x-ray cross sections (σ_Lβ1_) to MO x-ray cross sections (σ_Lβ1_/σ_MO_ ) and Lβ_2_ x-ray cross sections (σ_Lβ2_) to MO x-ray cross sections (σ_Lβ2_/σ_MO_) as a function of the incident energy, for charge states Xe^17+^, Xe^19+^ and Xe^23+^. Obviously, these ratios are increasing with increasing indent energy.

In addition, two ‘bumps’ located on the low energy side of the Xe^q+^ L x-ray lines are observed in our experiment, denoted by R_1_ and R_2_ in Fig. 1. Usually, an inner shell vacancy decays either through a radiative or a non-radiative transition. However, as suggested by T. Åberg *et al*., it may also decays by the simultaneous emission of a photon and an electron. The emitted photon has energy slightly lower than that of a characteristic x-ray photon and forms low energy peaks. This process is called the Radiative Auger Effect (RAE)^33^,^34^. In our results, peaks R_1_ and R_2_ originate from the RAE, as they are located on the low energy side of the projectile L-shell x-ray peaks.

**Figure 5:**
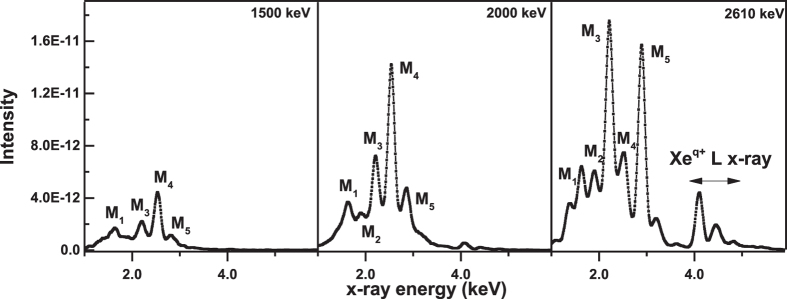
X-ray spectra from collisions of Xe^29+^ ions with Zn target at 1500 keV, 2000 keV and 2610 keV. M_1_: 4p_1/2_ → 3d_5/2_; M_2_: 4p_3/2_ → 3d_3/2_; M_3_: 4f_5/2_ → 3d_5/2_; M_4_: 4f_7/2_ → 3d_3/2_; M_5_: 4d_5/2_ → 3p_3/2_. The spectra have been normalized by the incident particle number.

Significantly, several new features are observed in the excited spectra for projectiles of charge state 29, as shown in Fig. 5.

Clearly, in the same energy range as the MO x-ray bands for the q < 29 cases, several narrow discrete lines are observed for an incident charge state of 29. It seems that the broad MO x-ray line ‘splits’ into several narrow lines when charge state q = 29, which is a phenomenon that has never before been reported in a quasi-molecular radiation study. Although the FWHMs of these narrow lines are similar to those of the characteristic lines Ll, Lα, Lβ_1_, Lβ_2_ and Lγ, they cannot be identified as characteristic lines of either target atoms or of the projectiles, as their centroid energies do not correspond to the characteristic x-rays of target atoms or the projectile ions. Taken together, these observations permit us to attribute these new narrow bands to the MO x-rays, as well. Hence, the molecular correlation diagram will once again be employed in the following analysis. In Fig. 3, as the inter-nuclear distance shrinks, the projectile orbital also evolves into molecular orbitals. Unlike Xe^q<29+^, Xe^29+^ ion itself has three 3d vacancies. That is, for the Xe^29+^+ Zn system, the predominant emission process for the MO x-rays is 4fδ → 3dδ. By contrast, the 3dσ-3dδ electron promotion process leads to projectiles carrying 2p vacancies. After separate, L-shell characteristic x-rays of the projectile ions emit.

Moreover, further analysis of the experimental data in Figs 1 and 5 using equations (1) and (2) reveals that, at the same incident energy, (i) The MO x-ray production cross sections induced by Xe^q<29+^ are of the same order of magnitude; (ii) Total σ_MO_(Xe^29+^) of all spectra M_n_ is approximately one order of magnitude smaller than σ_MO_(Xe^q<29+^) (see Table 1). These findings indicate that q influences not only the MO x-ray spectral profile and its origin, but also its production cross sections. Importantly, the production cross sections are closely related to the effective geometric range r_eff_ where MO transition may occur; see the shaded area in Fig. 3. Specifically, the relationship between them can be expressed in the following form^35^:


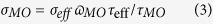


where, *σ*_*eff*_* = πr*_*eff*_^*2*^ and *τ*_*eff*_* = r*_*eff*_*/v*.

Then, the effective geometric range induced by Xe^q+^ ions is


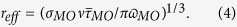


Where, _MO_ describes the average lifetime of a vacancy in the quasi-molecule; _MO_ represents an average fluorescence yield and the production cross sections σ_MO_ (see Table 1) is extracted from the experimental data in Figs 1 and 5 using equations (1) and (2).

To look for the further reason causing MO x-ray division, the r_eff_ for q <29 and q = 29 is calculated using equation (4), respectively.

Taking 2000 keV Xe^21+^+ Zn as an example, we have v = 1.71 × 10^8^ cm/s, σ_MO_ = 1.02 × 10^−22^ cm^2^ (from Table 1), _MO_ = 3.6 × 10^−2^ and 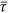
_MO_ = 3 × 10^−16^ s^36^,^37^,^38^, consequently, r_eff_ ≈ 0.33 × 10^−9^ cm. As we know, for the filling of a MO vacancy to occur during an interaction, the MO vacancy lifetime 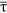
_MO_ should be comparable to the effective collision time τ_eff_, evaluated as r_eff_/v. However, in our experiment, 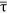
_MO_ is about one order of magnitude larger than τ_eff_. Two factors may lead to this discrepancy. (i) A straight-line trajectory, which results in the shortest possible route and time, is assumed in our calculation of r_eff_ and τ_eff_. In a real event, however, Coulomb (97) deflection plays an important role, especially in a slow bombardment^1^, and this effect will significantly increase the interaction time and the effective range. (ii) 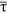
_MO_ is appreciably larger in a UA than in a quasi-molecule^35^. In Eq. (4), the average lifetime of a Po 3d vacancy is employed as there is no way to know the true 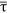
_MO_ value in a quasi-molecule. Hence, in a real impact event, the difference between the two values should be much smaller than the calculated ones. It is clear that the present experiment conditions satisfy the space and time requirements for MO transition to occur. In addition, the EPEs, the transition configurations, the production cross sections σ_MO_ and the effective ranges r’_eff_ for q = 29 are listed in Table 1.

Table 1 tells us that, at the same incident energy, (i) The total σ_MO_(Xe^29+^) for all peaks M_n_ is about one order of magnitude smaller than σ_MO_(Xe^q<29+^); (ii) The r’_eff_ corresponding to each narrow spectrum M_n_ is lower than that of peak M by approximately a factor of 5 and the total r’_eff_ for Xe^29+^ is very similar to r_eff_ for Xe^21+^. For comparison, the effective ranges (r’_eff_ and r_eff_) for both peaks M_4_ and M at 2000 keV are roughly indicated in Fig. 3. From the molecular correlation diagram, it is evident that a smaller r_eff_ can induce a narrower x-ray energy variation, i.e., a smaller FWHM. According to the above analysis, it is very easy to understand why, in our experiment, a broad MO x-ray band is observed for Xe^q<29+^, whereas several discrete peaks are observed for Xe^29+^. The small MO transition effective range r’_eff_ for Xe^29+^ ions is the key to explaining these experimental results. Moreover, our measured MO x-ray cross sections show a remarkably close match with this requirement (see Table 1). However, in this field, although some relevant experimental results have been reported, quantitative calculations of spectral structure and intensity are still sorely lacking, especially for multi-electron systems. To date, theoretical efforts have been primarily focused on few-electron quasi-molecules. Hence, with regard to our result, the question of why the effective range r’_eff_ is small for Xe^29+^ remains open to theoretical investigation.

## Conclusion

In summary, the non-characteristic and characteristic x-ray spectra measured in collisions of 1500–3500 keV Xe^q+^ (q = 12–29) ions with solid target Zn are investigated in detail. In the study, two different electron-vacancy exchange processes, namely, electron promotion and MO transition, corresponding to the characteristic x-rays and MO x-rays emissions respectively, are observed simultaneously in the same formation of the quasi-molecule Xe-Zn. Both the yields of characteristic x-rays and MO x-rays show a marked rise with an increase in the incident energy and the former grows faster than the latter. Meanwhile, the FWHMs of the MO band are measured and found to be proportional to the square root of the projectile velocity when the projectile charge states q <29. Moreover, the present work finds very striking difference appeared in MO x-ray spectra produced by Xe^q<29+^ and Xe^29+^ ions. Several narrow discrete lines are observed for an incident charge state 29, whereas a broad x-ray band is observed for Xe^q<29+^. It seems that the broad MO X-ray line ‘splits’ into several narrow lines as the charge state q = 29. This finding puts forward a new question of how the MO transition depends on the projectile charge state. Although the present work tries to answer the question and gives some explanations, more experiments and, in particular, deeper theoretical investigations are definitely necessary for its full understanding.

## Additional Information

**How to cite this article**: Guo, Y. *et al*. The continuous and discrete molecular orbital x-ray bands from Xe^q+^ (12 ≤ q ≤ 29) + Zn collisions. *Sci. Rep.* **6**, 30644; doi: 10.1038/srep30644 (2016).

## Figures and Tables

**Table 1:** Summary of peak M_n_ transition configurations^24^, EPEs, σ_MO_ values and r’_eff_ values for Xe^29+^+ Zn.

q	peak	IE (keV) EPE (keV)		1500		2000		2610 (3500)	
		Exp.	The.	σ_MO_ (b)	r’_eff_ (Å)	σ_MO_ (b)	r’_eff_ (Å)	σ_MO_ (b)	r’_eff_ (Å)
29	M_1_ (4p_1/2_ → 3d_5/2_)	1.75	1.83	7.05	0.80 (−2)	13.3	1.04 (−2)	18.5	1.21 (−2)
29	M_2_ (4p_3/2_ → 3d_3/2_)	2.03	2.10	1.05	0.42 (−2)	3.88	0.69 (−2)	9.35	0.96 (−2)
29	M_3_ (4f_5/2_ → 3d_5/2_)	2.40	2.50	0.28	0.44 (−2)	0.98	0.70 (−2)	1.93	0.92 (−2)
29	M_4_ (4f_7/2_ → 3d_3/2_)	2.70	2.79	0.60	0.56 (−2)	1.39	0.78 (−2)	0.95	0.72 (−2)
29	M_5_ (4d_5/2_ → 3p_3/2_)	2.90	2.82	0.27	0.42 (−2)	0.92	0.66 (−2)	1.78	0.86 (−2)
Total σ_MO_ or r’_eff_		—	—	9.25	0.264 (−1)	20.47	0.387 (−1)	32.54	0.467 (−1)
21	M (4f_7/2_ → 3d_3/2_)	2.70	2.79	67.5	0.27 (−1)	102	0.33 (−1)	242	0.48 (−1)

For comparison, the values for the spectrum M induced by Xe^21+^ and the total σ_MO_ and total r’_eff_ for Xe^29+^ are also listed. Numbers in parentheses indicate powers of ten. The errors of x-ray production cross sections are about 15%, including statistical and systematic errors.
